# Guidelines, Frameworks, and Trainings on Lived Experience and Caregiver Engagement in Research: Common Threads With Specific Considerations for Specific Subpopulations

**DOI:** 10.1111/hex.70772

**Published:** 2026-07-21

**Authors:** Lisa D. Hawke, Hannah Burgé Luviano, Kylie Payne, Thalia Phi, Hannah Monroe, Connie Putterman, Melissa Hiebert, Susan Conway, Elizabeth Hollingdrake, Aloha Narajos, Lina Chiuccariello

**Affiliations:** ^1^ Centre for Addiction and Mental Health Toronto Ontario Canada; ^2^ University of Toronto Toronto Ontario Canada

Individuals with lived and living experience of health challenges and family members/caregivers are experts of their experience and are key knowledge keepers in research about their needs [1]. Increasingly, they are engaged as advisors, co‐researchers, or equal partners in research projects. In these roles, they advise, collaborate on, co‐produce, co‐create, or lead many aspects of research projects. Possible contributions span the research lifecycle, from priority setting to knowledge mobilisation. While increasingly common across the health research sector, engagement may be particularly important in mental health and substance use health research as a response to the injustices, stigma, oppression, and coercive treatment specific to this area of care [2]. Engagement guidelines, frameworks, and trainings are required, helping to ensure that engagement is accessible, psychologically safe, and ethical for those engaged, while placing them in roles as partners to the research that is generated.

There are a range of guidelines, frameworks, and trainings to support lived experience and family/caregiver engagement in health research, inclusive of a range of sensitive health issues such as mental health and substance use health. Examples include the Patient Engagement in Research conceptual framework and accompanying psychometric scale [3, 4], materials offered by the Canadian federal funding agency [5], and guidance from organisations such as the Patient‐Centred Outcomes Research Institute in the United States [6] and the European Patient Engagement Resource Centre [7]. In addition to these general bodies of work, literature reviews and writings continue to proliferate on engagement among specific subpopulations, such as engagement among youth [8], veterans [9], those with psychosis [10], those in forensic settings [11], and those with neurodevelopmental conditions [12], with many resources specific to sensitive areas of health research. A 2019 scoping review identified 65 frameworks to support aspects of lived experience/patient engagement in research [13], and more have emerged since that time.

As a whole, the range of guidelines, frameworks, and trainings available converge on a number of notions. These include, for example, truly valuing the importance of lived expertise, being authentic and intentional in engagement approaches, balancing power dynamics, providing clear communication and accessibility‐based approaches, using a trauma‐informed approach to engagement, and formally recognising and compensating lived experience contributions, among others. A literature review of the barriers and facilitators to engagement across subpopulations identified many commonalities, highlighting, for example, the importance of inclusivity, strong communication practices, clarifying expectations, navigating operational challenges, and ensuring equitable power sharing [14]. These findings suggest that lived experience and family/caregiver engagement can be guided by a broad set of principles shared across a wide range of populations. However, the literature often breaks down engagement recommendations by subpopulation, suggesting differences that are not always described.

In light of the substantial similarities in engagement approaches across subpopulations, the question then becomes, ‘What are the differences?’ If multiple parallel literatures continue to proliferate describing and discussing highly similar engagement approaches for different subpopulations, the differences between them merit highlighting.

The Centre for Addiction and Mental Health, in Toronto, Canada, is a tertiary care teaching and research hospital that has prioritised engaging individuals with lived experience and family/caregivers throughout research and that aims to apply best practices for authentic engagement across the populations served. In May 2026, we conducted a 1‐day institutional Research Engagement Day. The theme of the event was the specificity of engagement across different subpopulations within the mental health and substance use health research sector. Attendees included staff, trainees, individuals with lived experience, family/caregivers, scientists, organising committee members, and keynote speakers. Throughout the course of the day, 105 attendees and presenters discussed specific considerations for engaging different subpopulations in research through oral and poster presentations and interactive activities. To stimulate dialog, we held a workshop activity in which attendees selected topic tables where they discussed specific populations of interest to them, with a focus on the specific best practices and needs of these communities in terms of authentic engagement. The subpopulations were selected by the attendees themselves, through a mid‐day polling activity. The tables focused on the specificity of engaging following subpopulations: (1) youth, (2) individuals with justice involvement, (3) individuals living with psychosis, (4) neurodivergent individuals, (5) individuals in rural and remote settings, (6) unhoused individuals, (7) 2SLGBTQ+ individuals, and (8) individuals from racialized communities. Field notes were taken, synthesised, and discussed after the event.

The content of the table discussions aligned with the findings in the literature: overarching engagement approaches and best practices for authentic, intentional, and meaningful engagement are essentially the same across subpopulations. What differs, though, is the amplification of certain considerations based on the characteristics of the subpopulation at hand.

Taking a closer look at the discussion content, we propose several examples of amplifications that were highlighted by attendees. Among youth, rapidly shifting developmental contexts and changing capacity was highlighted, together with the need for adaptation. Among individuals with justice‐system involvement, issues of power become particularly prominent. Among those living with psychosis, the diversity and unpredictability of symptom cycles was highlighted, which can impact engagement experiences. In neurodivergent populations, accessibility and inclusion were highlighted as particularly salient factors that must be explicitly addressed. For those living in rural and remote communities, there is an amplification of access barriers in light of community isolation, which must be addressed when recruiting this subpopulation into engagement roles. For those who are unhoused, the need to provide basic essentials like food and warmth was unique, together with the need to meet people where they are at. For 2SLGBTQ+ communities, the importance of validating unique experiences and providing a safe space is particularly salient; issues of cultural safety and Indigenous engagement approaches are especially important among Two‐Spirited individuals in First Nations, Inuit & Métis populations. In racialized communities, the inherent diversity within this broad group was highlighted, which might suggest the need to engage multiple voices for broad representation. Across groups, it was recognised that there are intersectionalities within and among them, that family considerations are important, and that stigma is omnipresent.

Importantly, all of these considerations are key practices in the engagement of any population across health conditions, within the mental health and substance use health research sector and beyond. Indeed, none are ever completely absent from the portrait of best practices in lived experience/patient, or caregiver/family engagement, regardless of the population in question. These needs are simply amplified among subpopulations with specific vulnerabilities around them.

We conclude that best practices for lived experience and caregiver engagement in research remain largely the same across conditions. The need, then, is for uniform guidelines, frameworks, and trainings that rigorously cover all best practices and set research teams up for authentic engagement. To optimise materials for specific subpopulations, it is important to highlight the best practices that are amplified in that subpopulation, reflecting on the *specificity* of engagement. Rather than duplicating our efforts with new guidelines, frameworks, and trainings specific to each subpopulation across areas of research on sensitive health issues, primary trainings might be supplemented with short modules that discuss which guidelines are amplified for specific subpopulations. While this work was conducted within the mental health and substance use health research sector, the homogeneity among best practice recommendations extends across health research sectors, and similar conclusions might be relevant to health research as a whole.

We call for a consolidation of guidelines, frameworks, and trainings on the best practices for engagement in health research that highlights amplifications for specific subpopulations rather than the generation of new materials for each subpopulation. In doing so, we can avoid duplication, focus the literature on the best available evidence, and streamline our workflows, while stimulating important dialogue on commonality versus specificity.

**Lived experience and caregiver engagement**. Individuals with lived experience and caregivers were involved in all aspects of the planning and execution of the Engagement Day event and the generation and discussion of the findings. They are co‐authors on this editorial.

## Author Contributions

**Lisa D. Hawke:** conceptualisation, writing – original draft, methodology, project administration, investigation, data curation. **Hannah Burgé Luviano:** conceptualisation, investigation, writing – review and editing, methodology, data curation. **Kylie Payne:** conceptualisation, investigation, methodology, writing – review and editing, data curation. **Thalia Phi:** conceptualisation, investigation, methodology, writing – review and editing, data curation. **Hannah Monroe:** conceptualisation, investigation, methodology, writing – review and editing, data curation. **Connie Putterman:** conceptualisation, investigation, methodology, writing – review and editing, data curation. **Melissa Hiebert:** conceptualisation, investigation, methodology, writing – review and editing, data curation. **Susan Conway:** conceptualisation, investigation, methodology, writing – review and editing, data curation. **Elizabeth Hollingdrake:** data curation, methodology, writing – review and editing, conceptualisation, investigation. **Aloha Narajos:** conceptualisation, investigation, methodology, writing – review and editing, data curation. **Lina Chiuccariello:** conceptualisation, investigation, methodology, writing – review and editing, data curation, resources.

## Conflicts of Interest

The authors declare no conflicts of interest.

## Data Availability

Data sharing is not applicable to this article as no datasets were generated or analysed during the current study.
